# Decadal spatiotemporal dynamics of surface water bodies in Zhengzhou, China: remote sensing monitoring and analysis of driving factors

**DOI:** 10.1038/s41598-025-32480-2

**Published:** 2025-12-22

**Authors:** Wei Song, Qingfeng Hu, Shilian Liu, Yaqian Hou

**Affiliations:** College of Surveying and Geo-informatics, North China University of Water Resources and Electric Power, Zhengzhou, 450046 Henan People’s Republic of China

**Keywords:** Environmental sciences, Environmental social sciences, Hydrology

## Abstract

Water is essential for life, human activities, and ecological balance. Understanding the spatiotemporal dynamics of urban water bodies is critical for sustainable water management, especially in fast-growing cities like Zhengzhou. This study investigates the evolution of water bodies in Zhengzhou from 2014 to 2023, using multi-seasonal Landsat 8 imagery and GIS analysis. Three water extraction methods-thresholding, random forest (RF), and a hybrid RF model incorporating spectral indices (NDWI, MNDWI, EWI) and GLCM-based texture features-were evaluated. The hybrid RF approach demonstrated the highest classification accuracy and was applied to generate seasonal water body maps for the target years. The results show a general increase in surface water area, with seasonal peaks in July–September, and spatial expansion mainly in the northern and northeastern regions. These changes are driven by both natural (e.g., precipitation) and anthropogenic (e.g., urban development, artificial lake construction) factors. Compared to previous studies, this research offers three key innovations: (1) a decade-long, seasonal-scale analysis of water dynamics; (2) a robust multi-feature classification framework for complex urban settings; and (3) an integrated interpretation of natural and human influences. The findings provide valuable references for water resource planning and urban ecological restoration, and the methods used may be transferable to similar inland cities under monsoon climates.

## Introduction

Water bodies are among the most vital natural resources on Earth, playing a critical role in maintaining ecosystem balance and directly influencing the sustainable development of human society. Changes in water bodies have become a focal point of global concern, and monitoring such changes is a key component of environmental science. This is particularly significant in the context of rapid urbanization, where water resource management and environmental protection face increasing challenges^1,2^. The decrease and increase of surface water are closely related to the local sustainable development, and monitoring its dynamics is important for the health of natural environment and sustainable economic development^3^. In recent years, remote sensing technology has been increasingly applied to monitor changes in water bodies. High-resolution satellite imagery enables the effective tracking of the spatiotemporal dynamics of water bodies. However, a notable challenge in applying optical remote sensing to urban environments is the presence of mixed pixels and high reflectance from impervious surfaces, which can interfere with the spectral separability of water bodies and reduce classification accuracy. Urban shadows, asphalt surfaces, and complex built-up textures often generate spectral confusion with small or narrow water bodies, making reliable extraction more difficult compared with natural landscapes.

Since the 1970s and 1980s, remote sensing technology has been increasingly employed for monitoring the dynamics of surface water bodies^4–8^. Compared with traditional survey methods, remote sensing offers higher efficiency and the capability to provide real-time and dynamic water body imagery^9^. The Landsat satellite series, launched by the United States Geological Survey (USGS) in 1972, has been in continuous operation for over five decades, accumulating a rich archive of remote sensing data. This includes data from Landsat 1–3 MSS, Landsat 4–5 TM, Landsat 7 ETM+, and Landsat 8 OLI. These satellites are characterized by short revisit intervals and the ability to repeatedly cover the same geographical area^10^, making them particularly suitable for analyzing the spatiotemporal changes of water bodies^11^.

In recent years, extensive research has been conducted both domestically and internationally on water body extraction using data from various remote sensing sensors. The principal methods currently employed include threshold-based approaches, classifier-based methods, and deep learning algorithms. Among them, threshold-based techniques are particularly represented by water index-based methods. Bartolucci et al.^12^ suggested that the near-infrared (NIR) band is the most effective for water body detection. Zhou Chenghu et al.^13^ identified a unique spectral relationship in TM imagery for water bodies-namely, (TM2 + TM3) > (TM4 + TM5)-which has proven particularly suitable for extracting water features in mountainous regions. Based on the normalized difference vegetation index (NDVI), McFeeters^14^ proposed the normalized difference water index (NDWI) model for water detection. Building on this, Xu Hanqiu^15^ introduced the modified normalized difference water index (MNDWI), which demonstrated higher accuracy in urban environments and effectively addressed the issue of shadow misclassification in water extraction. Yan Pei et al.^16^ developed the enhanced water index (EWI), which, when integrated with GIS technology, allowed for effective extraction of hydrological features in semi-arid regions. Compared to other methods, water index-based approaches are characterized by simplicity, high efficiency, and relatively high accuracy^17,18^. However, threshold-based methods also exhibit certain limitations. These approaches rely solely on the spectral characteristics of water bodies, and are susceptible to errors caused by mixed pixels, leading to inaccurate delineation of water boundaries and poor morphological representation. Determining an appropriate threshold requires careful consideration of data-specific factors, such as band selection and threshold calibration techniques. This process often demands extensive experimentation and is prone to manual intervention, limiting automation. Furthermore, threshold-based methods typically exhibit poor adaptability to complex scenarios or scenes with significant spectral variability. Their performance may degrade in such cases, often requiring scene-specific design of spectral rules or water indices, thereby limiting generalization capability.

With the continuous advancement of machine learning, a variety of classification algorithms have been developed and widely applied in water body extraction. Classifier-based methods, grounded in statistical learning theory, operate by training a model using a set of labeled sample data to learn the relationships and distinguishing features between classes, which can then be applied to classify unseen data. Compared to threshold-based approaches, classifier methods exhibit greater adaptability, generalization capability, and automation, thereby improving both the accuracy and efficiency of water body extraction. Paul et al.^19^ employed support vector machines (SVM), K-means clustering, and random forest (RF) algorithms to extract water bodies, among which SVM demonstrated superior performance. Chen Jingbo et al.^20^ analyzed the spectral and spatial characteristics of urban water bodies and constructed a knowledge-based decision tree, achieving an extraction accuracy of 86% for urban water bodies. Chen Yanhua et al.^21^ utilized Landsat 5 TM imagery and the C4.5 decision tree algorithm to extract various land cover types-such as water bodies, paddy fields, and wetlands-in the upper reaches of the Han River, achieving user accuracies of 73%, 83%, and 77%, respectively.

With the rapid development of artificial intelligence technologies, researchers worldwide have explored the application of deep learning for water body extraction from remote sensing imagery. Weng et al.^22^ proposed a separable residual segmentation network (SR-SegNet) that integrates residual convolutional structures to extract deep water body features while effectively preventing gradient vanishing during network training. As deep learning networks become increasingly deeper and more complex, such residual structures have been widely adopted in numerous remote sensing studies involving deep learning^23–26^. Yu et al.^27^ pioneered the combination of convolutional neural networks (CNN) with logistic regression classifiers, introducing a deep learning framework for hierarchical spatial–spectral feature extraction of water bodies, which captures the spatial frequency characteristics of water features at different depths from raw imagery. Long et al.^28^ introduced the fully convolutional network (FCN), marking the first application of deep convolutional networks to image semantic segmentation tasks. Building upon FCN, a series of advanced architectures such as U-Net^29^, SegNet^30^, DeepLab^31^, and PSPNet^32^ have been proposed and further optimized for specific applications. These semantic segmentation models have also been extensively adopted in the field of remote sensing for automated water body extraction, representing the forefront of current research. While deep learning approaches offer a high degree of automation in water body extraction from remote sensing images, their performance heavily depends on the availability of large, high-quality annotated datasets. This limitation restricts their generalizability in large-scale applications^29^. Additionally, training deep learning models requires substantial data volume and computational resources, resulting in long training times and high computational costs^12^.

Zhengzhou, a fast-growing megacity and the core of the Central Plains Urban Agglomeration, is undergoing rapid urban expansion, ecological restructuring, and intensive hydrological modifications. As a key transitional zone between the Yellow River and Huai River basins, the city holds strategic ecological importance for regional water regulation and flood mitigation. Rapid urbanization has reshaped its natural hydrological patterns, making Zhengzhou an ideal case for investigating long-term and seasonal water body evolution in a highly stressed urban environment. Understanding the spatiotemporal dynamics of Zhengzhou’s water bodies and their driving mechanisms is crucial for supporting sustainable water management and ecological restoration initiatives.

This study takes Zhengzhou City as the research area and utilizes Landsat 8 satellite imagery as the primary data source to extract the spatiotemporal distribution of water bodies over the past decade. The performance of water body extraction methods was comparatively analyzed, including empirically thresholded water indices (NDWI, MNDWI, and EWI) and random forest classifiers integrating water indices and image features. Based on the accuracy evaluation, the random forest method incorporating both water indices and spectral–textural features was ultimately selected to extract seasonal water body distributions for the years 2014, 2017, 2020, and 2023. Using the extracted data, this study investigates both intra-annual and inter-annual spatiotemporal variation patterns of water bodies in Zhengzhou over the last ten years. It further identifies the seasonal and long-term trends in water surface area changes, explores the spatial distribution of water body expansion or contraction, and analyzes the potential driving factors. The results provide scientific support for water resource management and environmental protection, promoting the sustainable utilization of urban water resources in Zhengzhou and beyond.

Using remote sensing techniques, this study extracted the water body distribution in Zhengzhou City from 2017 to 2023 and analyzed the spatiotemporal dynamics and driving factors of water body changes. The main conclusions are as follows:

1. From 2014 to 2023, the total water body area in Zhengzhou exhibited an overall increasing trend. Intra-annual fluctuations were observed, with larger water surface areas typically occurring between July and September.
2. The spatial centroid of water bodies showed a general northwest-to-northeast migration pattern. This shift is primarily attributed to the intensive urban development and implementation of water system planning in areas such as Zhengdong New District, where the construction of artificial lakes, wetland landscapes, and river channel improvements significantly expanded the water body coverage in the northeastern part of the city.
3. The spatial evolution of water bodies in Zhengzhou from 2014 to 2023 was driven by the combined effects of both natural and anthropogenic factors.

This study differs from previous research on urban water dynamics in several important ways.

1. It investigates the seasonal-scale evolution of urban water bodies over a decadal period (2014–2023) using multi-temporal Landsat imagery, providing finer temporal resolution than typical annual analyses.

(2) It integrates spectral indices and texture-based features in a Random Forest framework, enhancing classification precision in complex urban environments.
(3) It examines the combined impacts of natural and anthropogenic drivers, linking water body changes with precipitation and land-use expansion.

These innovations offer new insights into the spatiotemporal evolution and driving mechanisms of water resources in rapidly urbanizing regions.

Moreover, the findings of this study have broader applicability beyond Zhengzhou. As a rapidly urbanizing inland city located in a semi-humid monsoon climate zone, Zhengzhou shares hydrological, climatic, and developmental characteristics with other major Chinese cities such as Xi’an and Wuhan. Therefore, the spatiotemporal patterns of water body change identified in this research, as well as the demonstrated effectiveness of the integrated remote-sensing approach, can provide valuable references for water resource management and ecological planning in similar urban environments. This highlights the potential transferability of the methodological framework and the relevance of the results to a wider range of fast-developing cities facing comparable hydrological and ecological pressures.

## Introduction of study area

### Study area

**Fig. 1:**
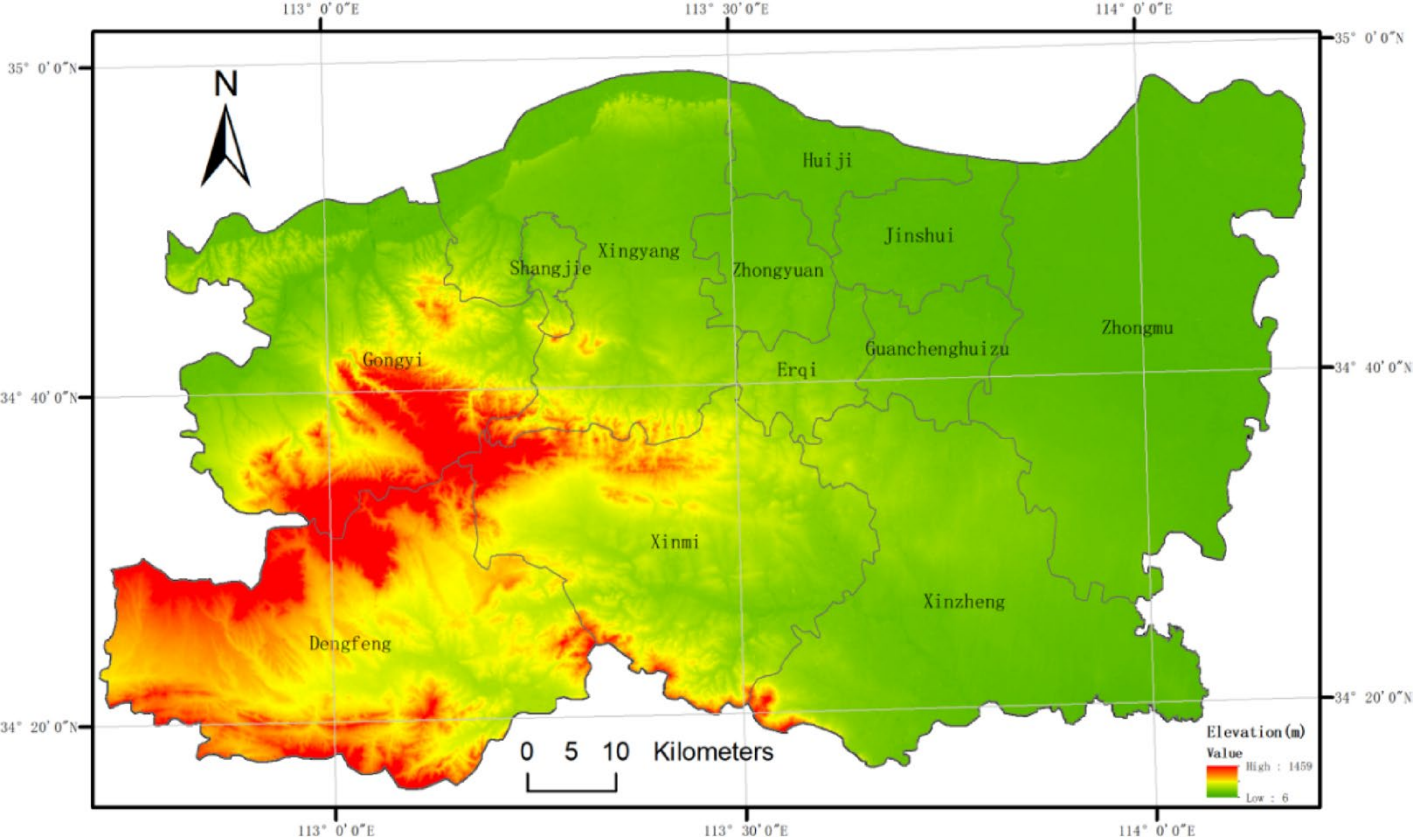
Location of the study area. The map shows the administrative boundary of Zhengzhou city, which is analyzed as a whole in this study rather than subdivided into specific districts. The analysis focuses on city-wide water body dynamics.

Zhengzhou, the capital city of Henan Province, is located in the middle and lower reaches of the Yellow River Plain, with geographic coordinates approximately at 34° 46′ N and 113° 39′ E, and a total administrative area of 7,446 square kilometers (Fig. 1). As the political, economic, and cultural center of Henan Province, Zhengzhou is one of the key cities in central China. The region is characterized by predominantly flat terrain, forming part of the classic Yellow River alluvial plain. Zhengzhou experiences a temperate monsoon climate with four distinct seasons, an annual average temperature of approximately 14 °C, and an annual precipitation ranging from 600 to 800 mm, with the majority of rainfall occurring from June to September. Hydrologically, Zhengzhou lies within the Huai River and Yellow River basins, with the Yellow River flowing through its territory. In addition to the main river systems, the city encompasses several significant water bodies, including tributaries of the Huai and Sha Rivers, as well as numerous man-made reservoirs and lakes. However, uneven spatiotemporal distribution of water resources and the rapid pace of urbanization have imposed considerable pressure on water availability and quality. Enhancing the management and protection of water resources, along with the rational utilization of aquatic ecosystems, has become a critical challenge for Zhengzhou’s sustainable development.

### Data

The remote sensing imagery used in this study was acquired from the online platform of the United States Geological Survey (USGS) (http://earthexplorer.usgs.gov/). The datasets consist of Landsat 8 imagery with a spatial resolution of 30 m, covering 16 scenes across four seasons in the years 2014, 2017, 2020, and 2023. All Landsat 8 OLI images used in this study were selected based on strict cloud cover requirements. Specifically, each scene had a cloud cover of less than 3%, ensuring that atmospheric disturbances caused by clouds had minimal influence on surface reflectance values. This low cloud threshold was necessary to maintain the reliability of water extraction, as cloud contamination and cloud shadows can lead to spectral distortions and misclassification in water body detection. Scenes with visible cloud contamination, cloud shadows, or haze were excluded from the analysis.The Landsat 8 imagery used in this study has a spatial resolution of 30 m, which may limit the identification of very small water bodies (< 900 m²). This constraint is acknowledged and considered in subsequent analyses. The vector boundary data for Zhengzhou City were obtained from the Geospatial Data Cloud (http://www.gscloud.cn/). Daily and monthly precipitation data for Zhengzhou from 2014 to 2023 were obtained from the China Meteorological Data Service Center (http://data.cma.cn), and used to qualitatively compare interannual water surface dynamics. Radiometric calibration, atmospheric correction, and spatial subsetting of each scene were performed using ENVI 5.6 software.

Landsat 8 remote sensing imagery offers high spatial resolution, multispectral capabilities, favorable temporal resolution, and global coverage, making it well-suited for water body extraction and spatiotemporal dynamic analysis. Moreover, its open-access policy and extensive historical data archive make Landsat 8 an ideal data source for studies on water body changes, providing a solid foundation for this research.

## Methods

**Fig. 2:**
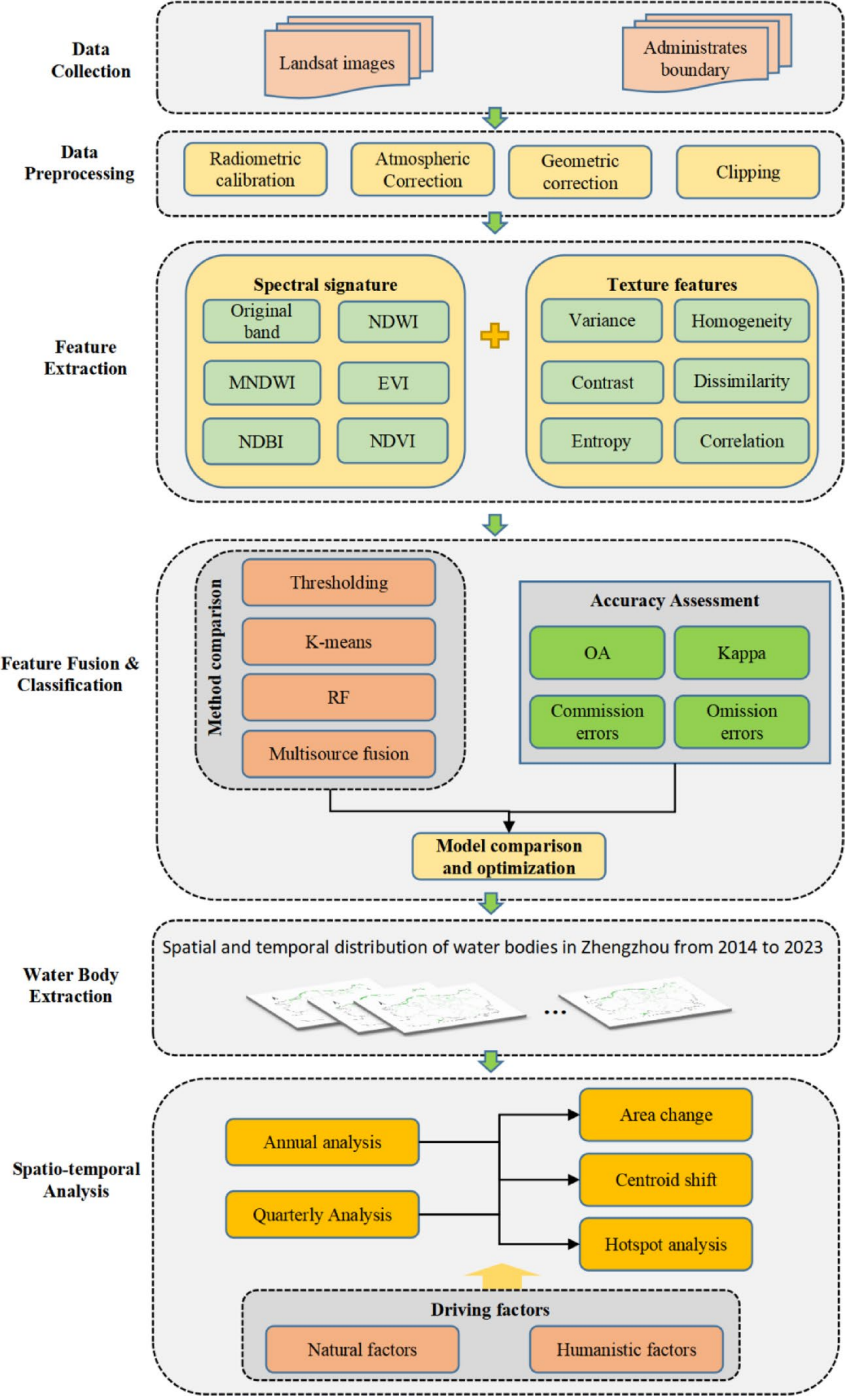
Research framework for remote sensing monitoring of water bodies.

The research framework of this study is illustrated in Fig. 2. First, Landsat imagery and vector administrative boundary data for Zhengzhou City were collected for multiple time periods. These images were then preprocessed and clipped to the study area. Subsequently, water body index features and texture features from the remote sensing imagery were extracted. Various water extraction methods were compared to identify the most suitable technique for delineating water bodies in Zhengzhou. Finally, GIS-based spatial analysis was employed to investigate the spatial–temporal dynamics and migration patterns of water bodies, and the driving factors of these changes were qualitatively analyzed.

### Data collection and preprocessing

To characterize the spatial and temporal dynamics of water bodies in Zhengzhou between 2014 and 2023, this study utilized multispectral imagery acquired from the Operational Land Imager (OLI) onboard the Landsat 8 satellite. A total of 16 cloud-free scenes (4 seasons × 4 years ) were selected, each representing spring (Q1), summer (Q2), autumn (Q3), and winter (Q4) conditions of the respective years.

#### Data source and selection criteria

All Landsat 8 OLI images were obtained from the United States Geological Survey (USGS) EarthExplorer platform. To ensure temporal consistency and minimize atmospheric interference, the images were selected according to the following criteria:

1. Cloud cover less than 3% within the study area to avoid cloud and cloud-shadow contamination;
2. Acquisition dates located in the mid-season period, and constrained within ± 10 days across different years to ensure seasonal comparability;
3. All scenes acquired from the same WRS-2 Path 124 / Row 36, which fully covers Zhengzhou, to maintain geometric consistency and avoid multi-scene mosaicking.

These criteria ensured that the selected images were radiometrically stable, seasonally representative, and suitable for long-term spatiotemporal analysis.

#### Radiometric and atmospheric correction

Radiometric calibration and atmospheric correction were applied to all Landsat 8 images to remove sensor-related noise and atmospheric scattering effects. These corrections ensure the spectral consistency of multi-temporal imagery and improve the spatial reliability of water body classification results across different acquisition dates.The following steps were followed:

- *Radiometric Calibration* Conversion of raw Digital Numbers (DN) to Top of Atmosphere (TOA) reflectance using metadata-based calibration coefficients.
- *Atmospheric Correction* The Fast Line-of-sight Atmospheric Analysis of Hypercubes (FLAASH) module within ENVI 5.3 was used to perform atmospheric correction, which accounts for water vapor, aerosol content, and adjacency effects.

#### Geometric correction and image registration

All images were resampled and co-registered to a common spatial reference system (WGS 1984 UTM Zone 50 N) using the Landsat reference scene from 2014 as the base image. The Root Mean Square Error (RMSE) of image alignment was maintained below 0.5 pixels.

To ensure geometric consistency across all years, we validated the co-registration accuracy using the Root Mean Square Error (RMSE) between each image and the 2014 reference scene. A set of evenly distributed tie points was automatically generated and manually checked to avoid mismatches over urban built-up areas. The RMSE was computed based on the residual distances of homologous points after geometric transformation. Only image registrations achieving RMSE < 0.5 pixels were accepted, which is consistent with the geometric precision recommended for multi-temporal Landsat analysis and ensures pixel-level comparability among the 16 scenes. This validation step guarantees that temporal changes in water bodies are not affected by geometric misalignment.

#### Cloud and shadow masking

Clouds and their shadows were masked using the CFMask (C Function of Mask) algorithm derived from Landsat’s Quality Assessment band. Manual inspection was performed to ensure the accuracy of cloud removal over urban water surfaces.

#### Subsetting and study area clipping

Each scene was clipped to the administrative boundary of Zhengzhou using a high-resolution vector. This step reduced computational load and ensured spatial focus on the target area.

### Spectral index calculation

To further enhance the separability between water and non-water features, several spectral indices were derived from the Landsat imagery, including the Normalized Difference Water Index (NDWI)^14^, the Modified Normalized Difference Water Index (MNDWI)^15^, the Normalized Difference Vegetation Index (NDVI), the Enhanced Vegetation Index (EVI), and the Normalized Difference Built-up Index (NDBI)^16,17^. Among these, NDWI and MNDWI were specifically employed to extract water bodies using the water index method. The water index approach is characterized by its simplicity, high efficiency, and applicability across diverse landscapes, making it one of the most practical methods for large-scale water body extraction.

#### Normalized difference water index (NDWI) and modified normalized difference water index (MNDWI)

NDWI and MNDWI are designed to enhance the spectral contrast between water bodies and built-up areas. They are defined as follows:

1 $$\:NDWI=\frac{Green-NIR}{Green+NIR}$$

2 $$\:MNDWI=\frac{Green-SWIR}{Green+SWIR}$$

where Green represents the reflectance of the green band, NIR represents the reflectance of the near-infrared band, and MIR represents the reflectance of the mid-infrared band.

#### Normalized difference vegetation index (NDVI)

The Normalized Difference Vegetation Index (NDVI) is one of the most widely used vegetation indices for monitoring vegetation cover and biomass. It enhances the contrast between vegetation and non-vegetated surfaces, which helps reduce the misclassification of vegetated land as water. NDVI is defined as:

3 $$\:NDVI=\frac{NIR-Red}{NIR+Red}$$

where NIR and Red represent the reflectance in the near-infrared and red bands, respectively. Higher NDVI values indicate denser and healthier vegetation, whereas water bodies typically yield low or negative NDVI values.

#### Enhanced vegetation index (EVI)

The Enhanced Vegetation Index (EVI) improves upon NDVI by optimizing the sensitivity to high biomass regions and minimizing atmospheric and soil background effects. It is particularly useful in urban and agricultural environments where spectral complexity is high. EVI is defined as:

4 $$\:EVI=G\cdot\:\frac{(NIR-Red)}{(NIR+{C}_{1}\cdot\:Red-{C}_{2}\cdot\:Blue+L)}$$

where NIR, Red, and Blue represent reflectance in the near-infrared, red, and blue bands, respectively; G is the gain factor; L is the canopy background adjustment factor; and C1, C2 are coefficients that correct for aerosol scattering influences. Typical constants are G = 2.5, L = 1, C1 = 6, and C2 = 7.5.

#### Normalized difference Built-up index (NDBI)

The Normalized Difference Built-up Index (NDBI) is designed to highlight built-up and impervious surfaces, thereby improving the discrimination between water bodies and urban areas. It is defined as:

5 $$\:NDBI=\frac{SWIR-NIR}{SWIR+NIR}$$

where SWIR is the shortwave infrared band reflectance and NIR is the near-infrared band reflectance. Water bodies typically exhibit negative NDBI values, whereas built-up areas and bare land generally yield higher positive values.

By combining these indices with the original spectral bands, the classification model can more effectively capture the spectral variability of different land cover types. This integration improves the recognition of water boundaries, wetland transition zones, and urban bare land, thereby enhancing the robustness of water body extraction in complex urban environments^17^.

#### Spectral and texture feature extraction

To enhance the separability between water and non-water surfaces, texture metrics were extracted from Landsat 8 imagery based on the Gray-Level Co-occurrence Matrix (GLCM) approach. The texture features included mean, variance, entropy, contrast, and homogeneity, which have been widely applied in surface water and land cover classification studies due to their ability to capture spatial heterogeneity and edge complexity.

All texture features were computed using a 3 × 3 moving window applied to Landsat bands 2–7 to represent multi-spectral spatial structure. The GLCM-based metrics were derived using the following parameters: pixel offset distance = 1, and calculation direction = 0°, 45°, 90°, and 135°, with the mean of all directions used as the final texture value.

These texture features were later combined with spectral indices (NDWI, MNDWI, and EWI) and original reflectance bands to construct the feature set for water classification using the Random Forest algorithm.

This approach has been proven effective for improving water body classification accuracy in medium-resolution imagery .

### Water body extraction methods

Traditionally, water body information derived from optical remote sensing data has been extracted primarily through manual visual interpretation. Although accurate, this approach is time-consuming, labor-intensive, and inefficient, making it unsuitable for large-scale applications. Current automated water extraction methods can generally be divided into threshold-based methods, classifier-based methods, object-oriented approaches, deep learning techniques, and other hybrid strategies^18^.

In this study, considering both practicality and efficiency, three representative classification models were selected: the threshold method, the Random Forest (RF) classifier, and the K-means clustering algorithm. To ensure reliable water body classification, the performance of different approaches was first compared, and the most suitable method was subsequently applied for water extraction in Zhengzhou.

#### Threshold method

The threshold method is a classical index-based approach for water body extraction, characterized by its simplicity and straightforward implementation. In this study, it was primarily applied to the Normalized Difference Water Index (NDWI) and the Modified NDWI (MNDWI). The underlying principle exploits the spectral characteristics of water, which typically exhibits higher reflectance in the green band and lower reflectance in the near-infrared (NIR) and shortwave infrared (SWIR) bands. By applying empirical or statistically determined thresholds, water pixels can be distinguished from non-water pixels^14,15^.

Although computationally efficient, the threshold method is susceptible to misclassification caused by vegetation, shadows, and high-reflectance built-up areas in urban or heterogeneous environments. Consequently, it is often used as a baseline approach or in combination with other classification methods.

#### Random forest (RF)

Random Forest is an ensemble learning algorithm that constructs multiple decision trees and determines the final class label through a majority voting strategy^19^. Previous studies have demonstrated its utility for water extraction. For example, Paul et al.^19^ compared Support Vector Machines (SVM), K-means, and RF, reporting that SVM achieved slightly better results. Chen Jingbo et al.^20^ constructed a knowledge-based decision tree by analyzing the spectral and spatial characteristics of urban water, achieving an accuracy of 86%. Chen Yanhua et al.^21^ applied Landsat-5 TM imagery with the C4.5 decision tree algorithm to extract rivers, paddy fields, and wetlands in the upper Han River basin, achieving user accuracies of 73%, 83%, and 77%, respectively.

In this study, RF was applied to (i) the original multispectral imagery, (ii) individual indices such as NDWI and MNDWI, and (iii) integrated feature sets that combined multiple indices (NDVI, EVI, NDBI, etc.). The advantages of RF include: (1) the ability to handle high-dimensional feature spaces and automatically assess feature importance; (2) robustness to noise and outliers; and (3) strong performance in complex urban environments, effectively reducing confusion between water, vegetation, and built-up areas.

#### K-means clustering

K-means clustering is an unsupervised classification method that partitions data into clusters by minimizing the within-cluster sum of squared errors (SSE). In water body extraction, K-means is often applied to NDWI and MNDWI distributions, where clustering centers are automatically generated to distinguish water from non-water regions^16^. As an unsupervised method, it does not require training samples and is therefore suitable for scenarios lacking prior information. However, its performance is sensitive to the number of clusters and initialization values, and in heterogeneous urban landscapes, its accuracy may be inferior to that of supervised methods such as RF.

In summary, by combining the strengths of the threshold method, Random Forest, and K-means clustering, this study provides a more comprehensive characterization of the spatial distribution of water bodies in Zhengzhou, while improving classification accuracy and reliability.

#### Comparative assessment of water extraction methods

In this study, the threshold-based approach, Random Forest (RF) classifier, and K-means clustering method were employed to extract water bodies from multi-seasonal Landsat 8 imagery. Each method presents distinct strengths and limitations in terms of computational efficiency, adaptability, and classification accuracy. The threshold method offers the highest computational efficiency because it relies solely on water index values such as NDWI or MNDWI; however, its performance is highly sensitive to scene-specific spectral variability and tends to produce misclassification in heterogeneous urban environments. The RF classifier provides the most accurate and robust results by integrating multi-source features-including spectral bands, water indices, and texture metrics-but it requires more computation time and labeled samples, making it comparatively more resource-demanding. K-means clustering operates in an unsupervised manner and does not rely on training data, which improves its transferability across scenes. Nevertheless, its spectral-distance-based clustering struggles to fully distinguish water from low-reflectance features such as shadows or asphalt, leading to reduced precision in complex urban areas. Overall, RF demonstrated the best balance between accuracy and stability, while thresholding and K-means serve as efficient but less adaptive alternatives in large-scale temporal analyses.

### Accuracy assessment of water body extraction

To systematically evaluate the classification accuracy of different water body extraction methods, this study employed several widely used accuracy metrics in remote sensing classification, namely Overall Accuracy (OA), the Kappa Coefficient (κ), Commission Error (CE), and Omission Error (OE)^33,34^.

Overall Accuracy (OA) is the most intuitive measure of classification accuracy, defined as the ratio of correctly classified samples to the total number of validation samples:

6 $$\:OA=\frac{\sum\:_{i=1}^{n}{X}_{ii}}{N}$$

where *Xii* represents the number of correctly classified samples in the diagonal of the confusion matrix, and *N* is the total number of samples. *OA* provides a straightforward estimate of the overall classification accuracy, but it may be biased in cases of class imbalance.

Kappa Coefficient (κ) measures the level of agreement between the classification results and the reference data, while accounting for agreement occurring by chance. It is calculated as:

7 $$\:\kappa\:=\frac{{P}_{o}-{P}_{e}}{1-{P}_{e}}$$

where *P*_*0*_ is the observed agreement (i.e., OA), and *P*_*e*_ is the expected agreement by chance. The Kappa coefficient ranges from − 1 to 1, with higher values indicating stronger agreement between classification and reference data.

Commission Error (CE) quantifies the proportion of pixels that were incorrectly assigned to a class, defined as:

8 $$\:CE=\frac{FP}{TP+FP}\times\:100\%$$

where *TP* is the number of true positives and *FP* is the number of false positives. A high *CE* indicates an overestimation problem, where non-water pixels are misclassified as water.

Omission Error (OE) refers to the proportion of pixels that actually belong to a given class but were not correctly identified in the classification. It is expressed as:

9 $$\:OE=\frac{FN}{TP+FN}\times\:100\%$$

where *FN* is the number of false negatives. A high *OE* reflects underestimation, meaning that water pixels were omitted from classification.

By jointly considering *OA*, *κ*, *CE*, and *OE*, classification accuracy can be comprehensively and quantitatively assessed, capturing both the overall performance and class-specific errors in water body extraction, including overestimation and underestimation.

### Methods for Spatiotemporal analysis of water bodies

#### Calculation of water body area

The area of water bodies can be calculated from the water regions extracted from remote sensing imagery for each year or season. Specifically, the number of pixels identified as water is counted, and the actual area is then derived by multiplying by the spatial resolution of the imagery. To ensure representativeness, time periods are carefully selected, typically by using data from the same season across multiple years to analyze seasonal variations, or by using annual datasets to investigate long-term dynamics.

#### Analysis of water body centroid migration

The analysis of water body centroid migration is performed based on water distribution data and Geographic Information System (GIS) techniques. The centroid location of the water body is calculated for each time period, and the spatiotemporal pattern of centroid shifts is analyzed across different seasons or years. This method helps to reveal the dynamic characteristics of water bodies, including expansion, contraction, and spatial redistribution trends^35,36^. In this study, centroid positions of water bodies were calculated for different seasons and years in Zhengzhou, enabling an assessment of the migration patterns and spatial evolution of water body distribution.

1. Calculation of water body centroid

The centroid position of a water body can be calculated using the following formula:

10 $$\:{C}_{x}=\frac{\sum\:_{i=1}^{n}{x}_{i}{A}_{i}}{\sum\:_{i=1}^{n}{A}_{i}},\hspace{1em}{C}_{y}=\frac{\sum\:_{i=1}^{n}{y}_{i}{A}_{i}}{\sum\:_{i=1}^{n}{A}_{i}}$$

where Cx and Cy represent the centroid coordinates of the water body in the horizontal and vertical directions, respectively; xi and yi are the coordinates of the i-th pixel within the water body region; and Ai is the area of the corresponding pixel. By computing the weighted average of all pixels within the water body region, the geometric centroid of the water body can be determined.

(2) Method for analyzing water body centroid migration

The analysis of water body centroid migration is conducted by comparing centroid positions across different temporal or seasonal intervals, thereby assessing the spatial dynamics of water body changes^35,36^. The degree of centroid migration is quantified by calculating the displacement of centroid positions between two time periods or seasons. The displacement is typically measured using the Euclidean distance:

11 $$\:D=\sqrt{({C}_{{x}_{2}}-{C}_{{x}_{1}}{)}^{2}+({C}_{{y}_{2}}-{C}_{{y}_{1}}{)}^{2}}$$

where (C_x1_,C_y1_) and (C_x2_,C_y2_) represent the centroid coordinates of the water body at two different time points. By computing the centroid displacements across time, both the migration rate and direction of water body dynamics can be derived.

(3) Limitations of centroid migration analysis

While centroid migration provides a useful summary metric for characterizing the directional shift of water bodies over time, it also has inherent limitations in complex urban environments. Because the centroid represents an area-weighted geometric mean, it may not fully capture spatial changes when water bodies are highly fragmented, narrow, or influenced by numerous small artificial features such as canals, ponds, and retention basins. In such cases, small positional shifts in individual patches can disproportionately affect the centroid location, potentially oversimplifying the underlying spatial heterogeneity. Therefore, centroid migration should be interpreted as a generalized indicator of overall spatial movement rather than a precise representation of all localized changes within the urban landscape.

#### Hotspot analysis

Hotspot analysis is primarily used to identify clusters or areas with significant spatial variation of a given phenomenon. In the context of spatiotemporal water body change analysis, hotspot analysis is frequently applied to reveal critical regions of water body dynamics, thereby providing a scientific basis for water resource management and ecological protection^37,38^.

Common hotspot analysis methods include the Getis-Ord Gi* statistic, spatial autocorrelation (Moran’s I), and Kernel Density Estimation (KDE). In this study, the Getis-Ord Gi* statistic was adopted to conduct hotspot analysis of water body changes.

The Getis-Ord Gi* statistic is one of the most widely used approaches for hotspot detection. It assesses the local spatial variation of each geographic unit and determines whether the unit exhibits significant spatial clustering^37,38^. The statistic is defined as:

12 $$\:{G}_{i}^{\ast\:}\left(d\right)=\frac{\sum\:_{j}{w}_{ij}\left(d\right)\hspace{0.17em}{x}_{j}}{\sum\:_{j}\hspace{0.17em}{x}_{j}}$$

where *Gi∗(d)* is the Gi* statistic value for geographic unit *i* at distance *d*, *w*_*ij*_*(d)* represents the spatial weight between unit *i* and unit *j*, and *x*_*j*_ denotes the attribute value of unit *j* (e.g., water body area change or change rate).

## Results and discussion

### Comparison of different water extraction methods

In this study, multiple approaches were employed for water body extraction in Zhengzhou, including the water index method (NDWI and MNDWI), the K-means clustering method (NDWI and MNDWI), the Random Forest (RF) method (original imagery), RF with original imagery combined with NDWI, RF with original imagery combined with MNDWI, RF with original imagery combined with NDWI and texture features, and RF with original imagery + NDWI + MNDWI + NDVI + EVI + NDBI + texture features. For the water index method, empirical thresholds (T) were applied to determine classification boundaries for NDWI and MNDWI.

The dataset was randomly divided into training and validation subsets with a 70:30 ratio, ensuring that no pixels used for training were included in the validation phase. This split design effectively minimizes sampling bias and prevents information leakage between datasets. Independent validation using external datasets (e.g., Sentinel-2 or Google Earth) was not conducted because the study focuses on temporal consistency within Landsat 8 imagery to maintain radiometric and geometric homogeneity across all years. Instead, a cross-validation strategy was adopted to ensure generalization performance. This approach is commonly used in large-scale water classification studies where consistent multi-temporal Landsat data are available.

Confusion matrices^33^ were constructed to assess classification accuracy. Four evaluation metrics-Overall Accuracy (OA), Kappa coefficient (κ), Commission Error (CE), and Omission Error (OE)-were employed to quantitatively assess the performance of each method. The accuracy statistics obtained from the experiments are summarized in Table 1.

As shown in Table 1, the RF method integrating original imagery with NDWI, MNDWI, NDVI, EVI, NDBI, and texture features achieved the highest overall accuracy, with the lowest CE and OE. The RF method combining original imagery with NDWI and texture features also produced excellent results, with OA exceeding 99% and both CE and OE remaining low. The RF method with original imagery + NDWI yielded the lowest CE but exhibited a relatively higher OE, indicating omission errors. By contrast, other classification approaches demonstrated lower OA, higher CE and OE, and failed to effectively distinguish water bodies from low-reflectance land cover types. Overall, the RF model integrating original imagery with NDWI, MNDWI, NDVI, EVI, NDBI, and texture features exhibited the best classification performance and was therefore adopted for the spatiotemporal analysis of water body dynamics in Zhengzhou.

**Table 1:** Accuracy assessment of water body extraction using different classification schemes.

Method	Features used	Overall accuracy(%)	Kappa coefficient	Commission (%)	Omission (%)
Threshold ( NDWI )	NDWI	98.80	0.91	1.09	15.33
Threshold ( MNDWI )	MNDWI	98.74	0.90	3.44	14.09
K-means ( NDWI )	NDWI	97.93	0.89	1.26	17.33
K-means ( MNDWI )	MNDWI	98.03	0.90	3.57	16.25
RF ( Original image )	Spectral bands	98.51	0.88	5.23	15.50
RF ( Original image + NDWI )	Spectral bands + NDWI	99.10	0.93	0.89	11.44
RF ( Original image + MNDWI )	Spectral bands + MNDWI	98.74	0.90	1.58	15.68
RF ( Original image + NDWI + Texture )	Spectral bands + NDWI + texture features	99.21	0.95	1.88	6.23
RF ( Original image + NDWI + MNDWI + NDVI + EVI + NDBI + Texture )	Spectral bands + multi-index integration	**99.38**	**0.95**	**1.33**	**5.40**

### Water body segmentation results and analysis

**Fig. 3:**
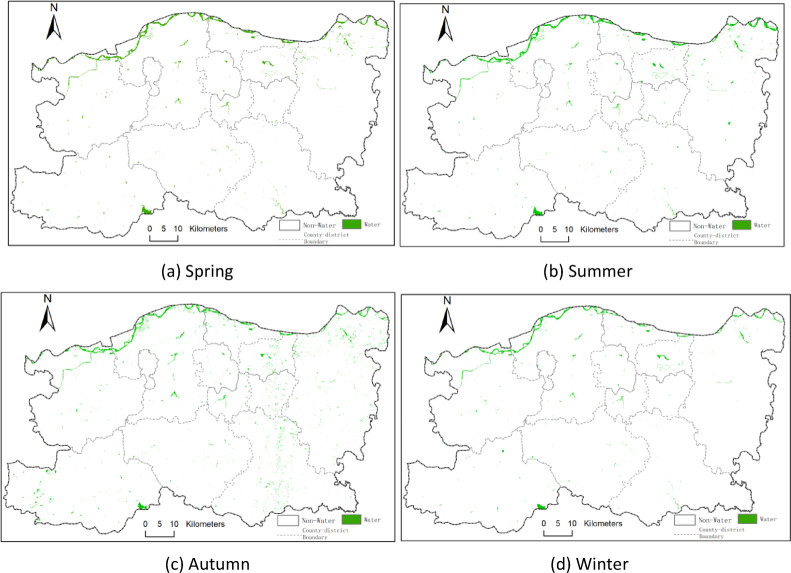
Seasonal distribution of surface water bodies in 2014 across Zhengzhou: (**a**) spring, (**b**) summer, (**c**) autumn, and (**d**) winter. Green areas represent classified water bodies based on Landsat 8 imagery; white areas represent non-water. All maps are at the same spatial scale with north oriented upwards. (**a)** Spring, (**b**) summer, (**c**) autumn, (**d**) winter.

**Fig. 4:**
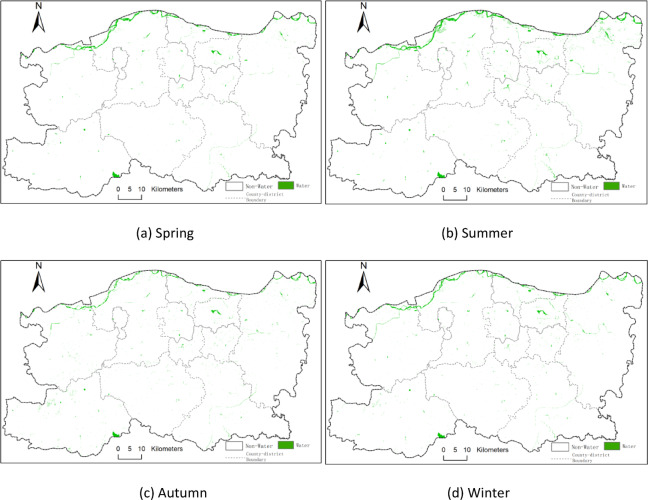
Seasonal distribution of surface water bodies in 2017 across Zhengzhou: (**a**) spring, (**b**) summer, (**c**) autumn, and (**d**) winter. Green areas represent classified water bodies based on Landsat 8 imagery; white areas represent non-water. All maps are at the same spatial scale with north oriented upwards. (**a**) Spring, (**b**) Summer, (**c**) Autumn, (**d**) Winter.

**Fig. 5:**
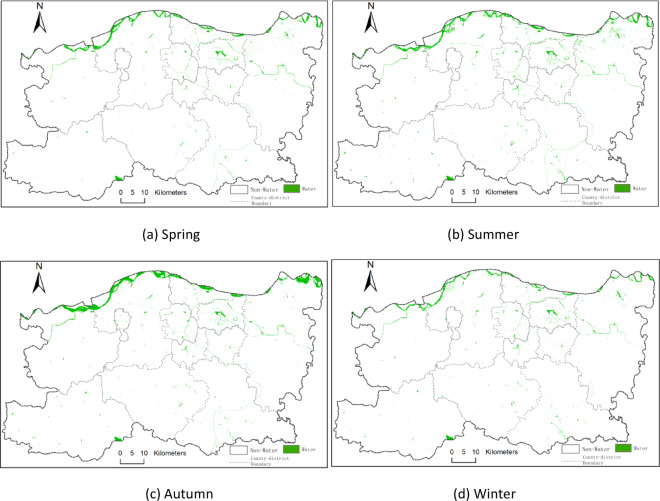
Seasonal distribution of surface water bodies in 2017 across Zhengzhou: (**a**) spring, (**b**) summer, (**c**) autumn, and (**d**) winter. Green areas represent classified water bodies based on Landsat 8 imagery; white areas represent non-water. All maps are at the same spatial scale with north oriented upwards. (**a**) Spring, (**b**) Summer, (**c**) Autumn, (**d**) Winter.

**Fig. 6:**
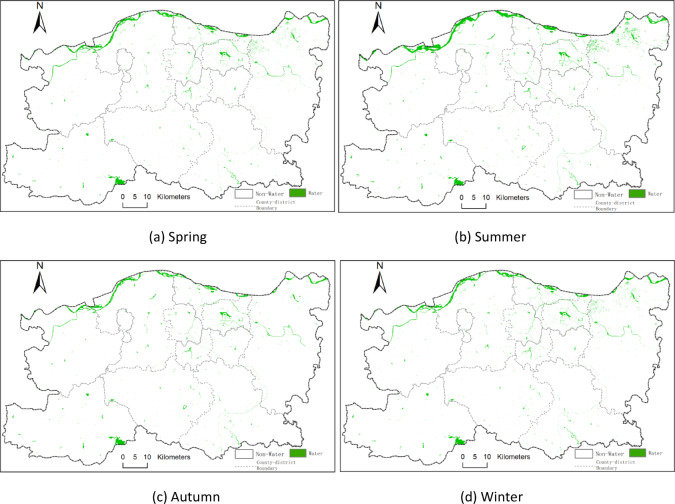
Seasonal distribution of surface water bodies in 2023 across Zhengzhou: (**a**) spring, (**b**) summer, (**c**) autumn, and (**d**) winter. Green areas represent classified water bodies based on Landsat 8 imagery; white areas represent non-water. All maps are at the same spatial scale with north oriented upwards. (**a**) Spring, (**b**) Summer, (**c**) Autumn, (**d**) Winter.

The study initially calculated several water body indices, including NDWI, MNDWI, and EWI, followed by water body extraction using the threshold method and K-means clustering. To further evaluate extraction performance, water body indices were fused with image features and classified using the Random Forest algorithm. The comparison results showed that the Random Forest model integrating NDWI with image and spatial texture features achieved superior classification accuracy. Figure 3 displays the seasonal water body distribution maps for 2014, while Figs. 4 and 5, and 6 illustrate those for 2017, 2020, and 2023, respectively. In these maps, green areas represent water bodies, and white areas denote non-water regions.

From Figs. 3, 4, 5 and 6, it can be observed that Zhengzhou is located at the junction of the Yellow River and Huai River basins. The main watercourse is the Yellow River, which primarily flows through the northern part of Zhengzhou. Tributaries such as the Sishui and Kuhe Rivers traverse from west to east across areas like Gongyi and Xingyang. Additionally, Huai River tributaries including the Jialu, Suoxu, and Jinshui Rivers represent smaller-scale water bodies. Other prominent water bodies include Xiliu Lake, Changzhuang Reservoir, and various artificial lakes.

Within the urban core, artificial water bodies dominate, such as Xiliu Lake and Jinshui River, which serve municipal water supply and ecological landscaping purposes. In peripheral areas (e.g., Gongyi City and Xingyang City), water resources rely heavily on Yellow River tributaries and reservoirs, such as the Heluo Wetland Park in Gongyi and the Suoxu River in Xingyang. This has resulted in a hydrological pattern characterized by “two zones with multiple tributaries.” The Baihe Reservoir is located in the southwestern part of Zhengzhou.

The seasonal water body maps presented in Figs. 4, 5 and 6 clearly illustrate how intra-annual hydrological variability shapes the overall spatiotemporal dynamics of surface water in Zhengzhou. Summer (Q2) consistently exhibits the largest water extents, which is attributable to the concentration of regional precipitation between June and September, resulting in substantial replenishment of rivers, reservoirs, and wetlands. By contrast, autumn (Q3) and winter (Q4) show markedly reduced water surface areas, reflecting seasonal drying, increased evaporation, and reduced inflow. Notably, the significant expansion observed in the summer of 2020 corresponds to the extreme rainfall event reported for August 2020, whereas the contraction observed in autumn 2023 aligns with below-average precipitation and elevated water demand. These seasonal patterns directly support the study’s core findings by demonstrating that both natural hydrometeorological processes and human water use exert strong, season-dependent influences on the spatial distribution and extent of water bodies across the city.

### Spatiotemporal analysis of water body changes in Zhengzhou

**Fig. 7:**
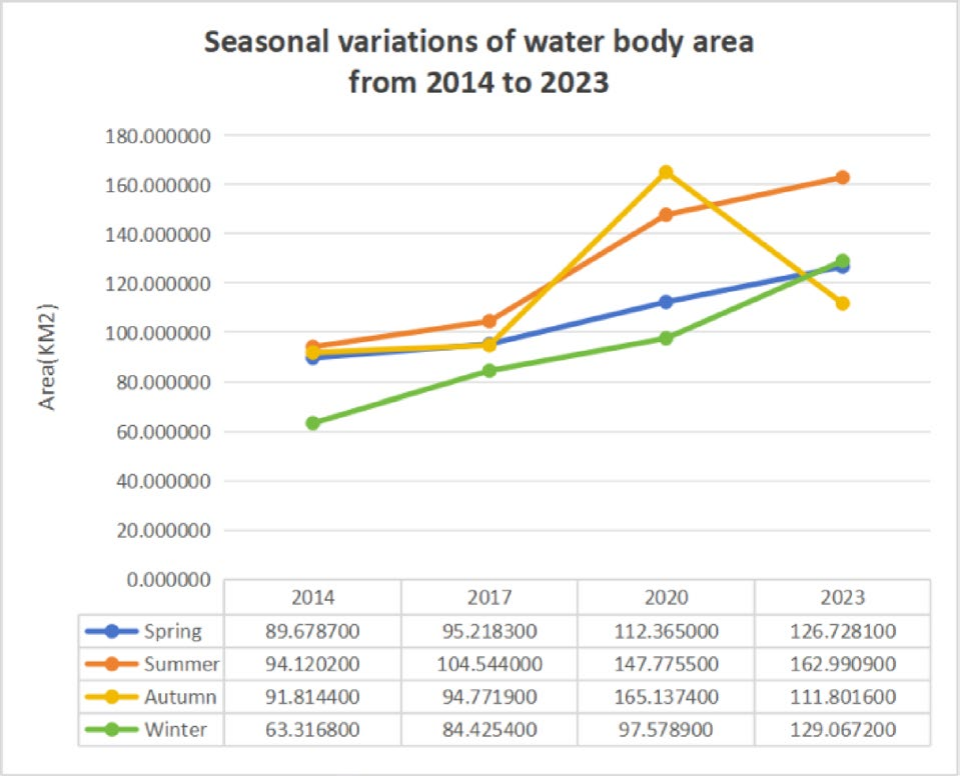
Seasonal variations of water body area in Zhengzhou from 2014 to 2023. Each data point represents a single Landsat 8 image acquired for the corresponding season and year. As only one scene per season was used, confidence intervals could not be computed; hence, the chart illustrates the temporal trend rather than statistical dispersion.

Based on remote sensing image analysis, the water surface areas of Zhengzhou City for all four seasons in the years 2014, 2017, 2020, and 2023 were calculated. These data were used to generate line charts showing the seasonal variations in water body extent across the selected years. Figure 7 presents the line graphs illustrating changes in Zhengzhou’s water bodies for each season. Specifically, the “Spring” graph shows water area variations from January to March across the four years; “Summer” depicts changes from April to June; “Autumn” illustrates data from July to September; and “Winter” shows variations from October to December.

As shown in Fig. 7, the water surface area in Zhengzhou is lowest during October to December, while the largest area is observed between July and September. Overall, the water body extent in Zhengzhou has shown an increasing trend over the past decade. A notable anomaly is the significant increase in water surface area during July to September 2020, which is primarily attributed to heavy rainfall in August of that year. The pronounced increase in water surface area observed in the summer of 2020 is closely associated with an extreme precipitation event that occurred in August of that year. Meteorological records indicate that rainfall across Zhengzhou reached 260–350 mm, exceeding the long-term average by 10–20% and causing substantial recharge of rivers, tributaries, reservoirs, and urban retention basins. This anomalously high precipitation led to widespread surface water accumulation, expansion of floodplains, and temporary inundation in low-lying alluvial areas, which is clearly reflected in the seasonal water extent maps and the peak values in Fig. 7. Conversely, the notable reduction in autumn 2023 corresponds to below-average rainfall and increased water consumption during a period of elevated agricultural and municipal demand. These contrasting events highlight the strong sensitivity of seasonal water body dynamics to short-term hydrometeorological fluctuations, and reinforce the conclusion that precipitation variability is a major driver of both intra-annual and inter-annual surface water changes in Zhengzhou. According to meteorological data, precipitation in Zhengzhou during August 2020 reached 260–350 mm, which is 10–20% higher than the historical average.

In contrast, a significant reduction in water area was observed in autumn 2023. This decline is mainly attributed to reduced precipitation and increased water demand. Specifically, rainfall in Zhengzhou during autumn 2023 was markedly below the multi-year average. For instance, in September 2023, the average precipitation across Henan Province was 161.5 mm, while the multi-year average was 79.3 mm-approximately twice the typical amount. This reduction in precipitation directly impacted water availability and replenishment of surface water bodies.

Furthermore, as a densely populated metropolitan area, Zhengzhou experiences high water demand. Despite various initiatives implemented to optimize water allocation and promote conservation, demand remains substantial. Increased water consumption across agricultural, industrial, and domestic sectors has further contributed to the observed reduction in surface water area.

As each seasonal water surface area was derived from a single Landsat 8 scene, statistical uncertainty (e.g., confidence intervals) could not be estimated. Therefore, the line chart in Fig. 7 represents the interannual variation trend rather than variance among samples. The trend still clearly shows a general increase in surface water extent, particularly during summer months (July–September).

**Table 2:** Quantitative analysis of seasonal water body area change (2014–2023).

Season	2014–2017 Change (km^2^)	2014–2017 Change (%)	2017–2020 Change (km^2^)	2017–2020 Change (%)	2020–2023 Change (km^2^)	2020–2023 Change (%)
Spring	5.54	0.073	17.15	0.227	14.36	0.19
Summer	10.42	0.138	43.23	0.571	15.22	0.201
Autumn	2.96	0.039	70.37	0.93	-53.34	-0.705
Winter	21.11	0.279	13.15	0.174	31.49	0.416

Table 2 summarizes the quantitative metrics of water area change during 2014–2023, including net and percentage changes for each period. Overall, the most significant increase occurred in summer and winter, while autumn showed a decline in 2020–2023. To further examine the relationship between hydrometeorological conditions and the temporal dynamics of water bodies, we compared the variations in annual precipitation with the observed water surface area during 2014–2023. The analysis revealed that years with higher precipitation, such as 2017 and 2020, generally corresponded to larger water surface areas, whereas drier years (e.g., 2014 and 2023) exhibited a noticeable decline in water extent. Although the limited number of temporal observations (four multi-seasonal Landsat scenes) precluded a robust statistical correlation analysis such as the Pearson test, the qualitative comparison clearly indicates a positive linkage between precipitation and surface water area. Future research using higher temporal resolution data, such as monthly composites from Sentinel or MODIS imagery, will allow for more rigorous statistical validation of this relationship.

### Centroid migration of water bodies in Zhengzhou

**Fig. 8:**
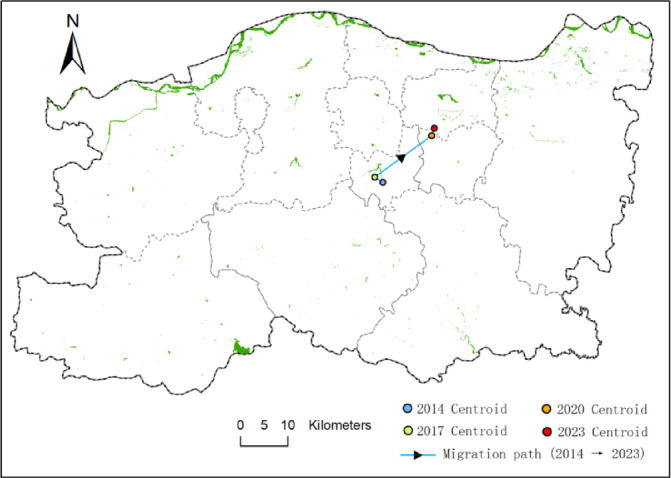
Trajectory of water body centroid migration in spring from 2014 to 2023. Each colored dot represents the centroid position for a given year, and the connecting arrows indicate the direction of temporal migration. The legend shows the year corresponding to each centroid, allowing for clear interpretation of spatial shifts over time.

After obtaining the seasonal water body distribution maps for Zhengzhou City across four years, this study calculated the centroid of water bodies for each season and year to investigate the migration patterns. As shown in Fig. 8, the centroid migration trajectory of water bodies in spring is illustrated, where each point represents the centroid of water body distribution for a specific year.

The figure reveals an overall northwest-to-northeast migration trend in the water body centroid of Zhengzhou. From 2014 to 2017, the centroid shifted predominantly northwest, with a cumulative displacement of 1.99 km and an average annual migration rate of 0.66 km/year. Between 2017 and 2020, the centroid exhibited a significant increase in migration, shifting toward the northeast with a cumulative displacement of 14.26 km and an annual average migration rate of 4.75 km/year. From 2020 to 2023, the migration slowed again, continuing northeastward with a cumulative displacement of 1.64 km and an average annual migration rate of 0.55 km/year.

These results indicate that the period 2017–2020 experienced the most dramatic spatial changes in water body distribution. This is likely attributable to the large-scale construction of artificial water bodies and ecological restoration projects in the Zhengdong New District.

**Fig. 9:**
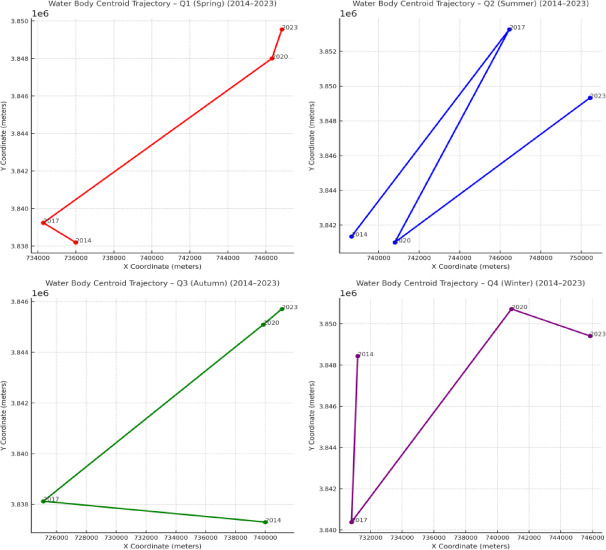
Quarterly trajectories of water body centroid migration in Zhengzhou from 2014 to 2023.

As shown in Fig. 9, the centroid migration trajectories of water bodies for the spring (Q1), summer (Q2), autumn (Q3), and winter (Q4) seasons from 2014 to 2023 are illustrated.Each subplot represents a distinct season (Q1 to Q4), and the centroid positions for each year are connected sequentially. The Y-axis denotes the geographic latitude (Y coordinate), and the X-axis denotes the longitude (X coordinate), with values projected in meters.

Notably, the centroids exhibit distinct migration patterns among seasons:

Spring (Q1) and summer (Q2) show larger spatial fluctuations, likely influenced by seasonal hydrological cycles and precipitation; Autumn (Q3) and winter (Q4) demonstrate more spatial consistency, indicating relative stability of perennial water bodies.

The overall northeastward trend across most seasons reflects long-term urban hydrological restructuring and expansion.

### Hotspot and coldspot analysis of water bodies

**Fig. 10:**
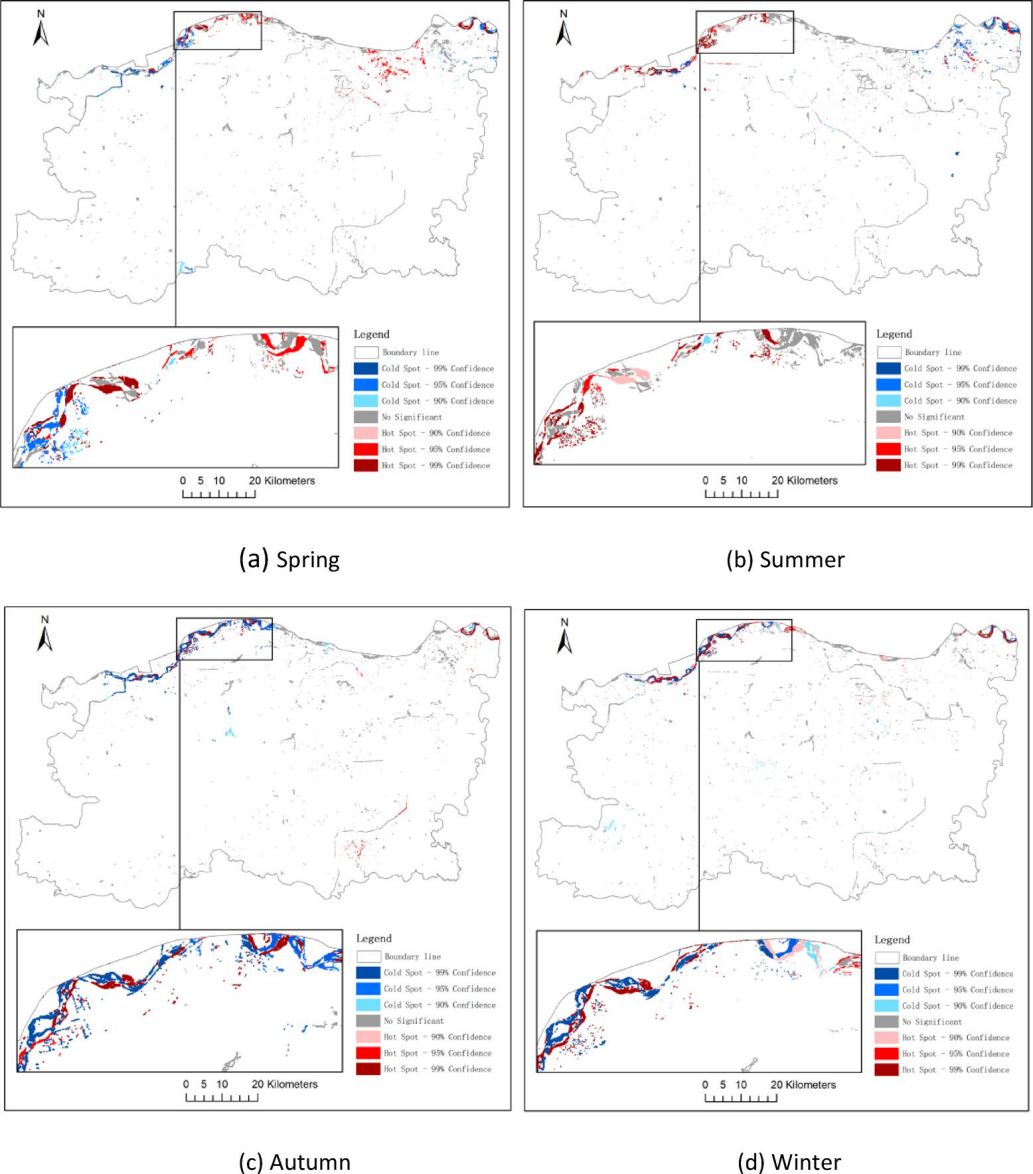
Hot and cold spot analysis of water area change from 2014 to 2017 (**a**: spring, **b**: summer, **c**: autumn, **d**: winter).

**Fig. 11:**
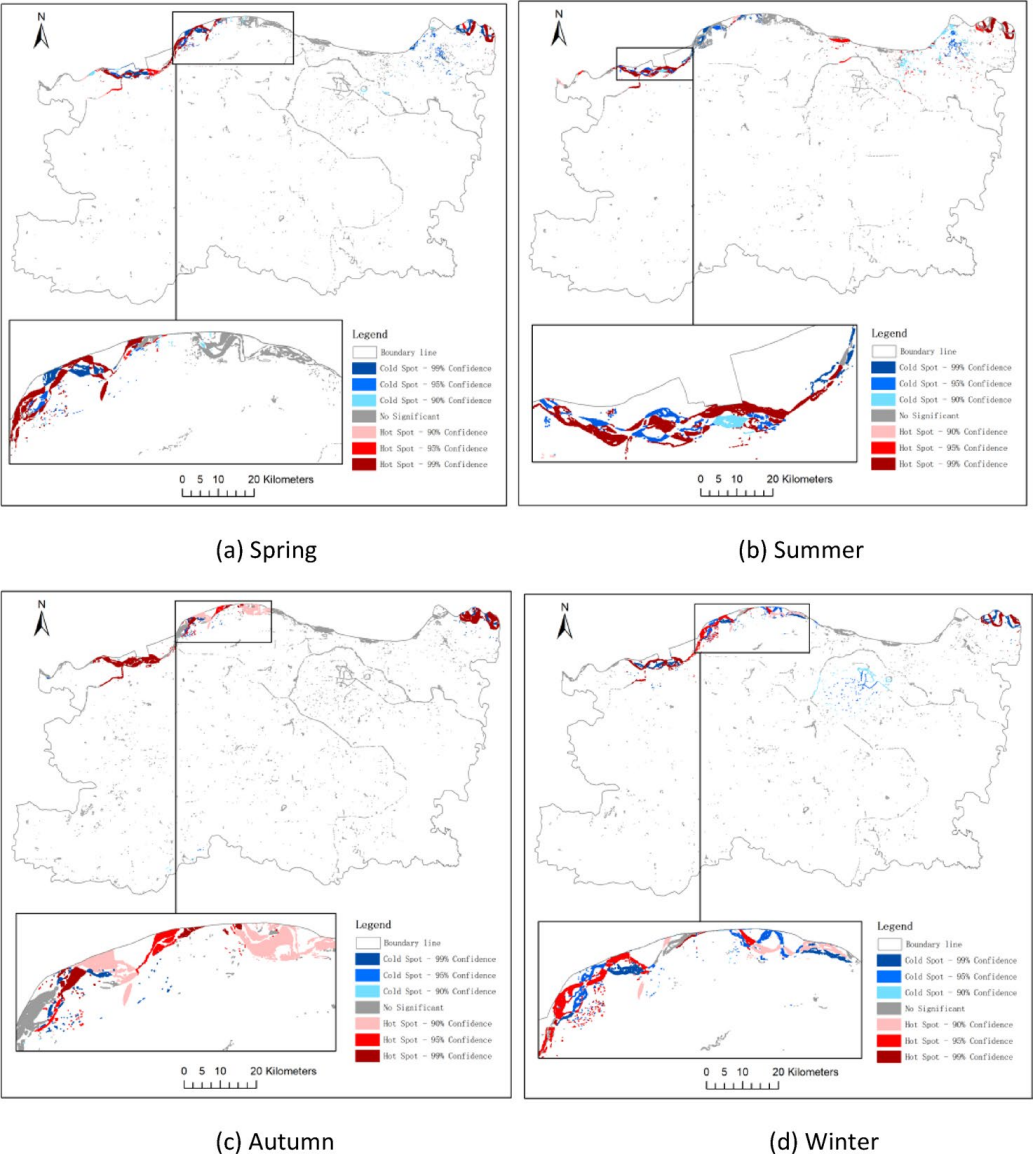
Hot and cold spot analysis of water area change from 2017 to 2020 (**a**: spring, **b**: summer, **c**: autumn, **d**: winter).

**Fig. 12:**
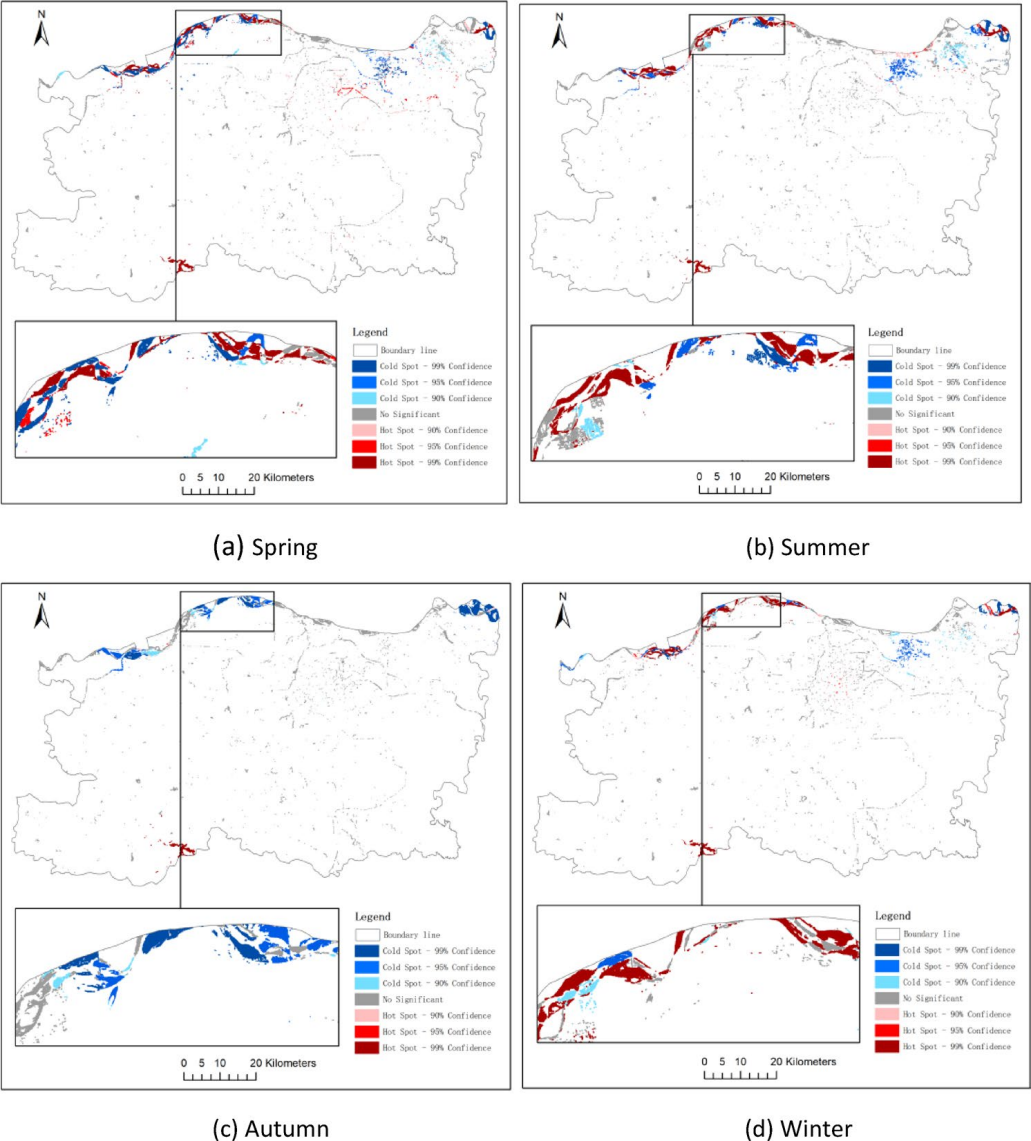
Hot and cold spot analysis of water area change from 2020 to 2023 (**a**: spring, **b**: summer, **c**: autumn, **d**: winter).

To minimize potential statistical bias arising from bipolar data distributions, areas with positive and negative changes in water surface were analyzed separately using the Getis-Ord Gi* statistic. This approach enabled a clear distinction between spatial clusters of significant expansion (“hot spots”) and contraction (“cold spots”). To further identify statistically significant clustering patterns of water surface dynamics, the Getis-Ord Gi* statistic was applied to areas of increased and decreased water bodies across three temporal intervals: 2014–2017, 2017–2020, and 2020–2023. The corresponding results are presented in Figs. 10 and 11, and 12.

1. Hot Spots (Gi* > 0).

Regions classified as hot spots represent spatial clusters where water area expansion is significantly concentrated. For instance: during 2020–2023, hotspots were mainly distributed in the eastern and northeastern parts of Zhengzhou, coinciding with the Yellow River wetlands and large-scale wetland restoration projects in the Zhengdong New District (Fig. 12a). In the same period, new hotspots also emerged in the southwestern Dengfeng area, which were likely driven by water management and ecological restoration initiatives, as well as favorable topographic and hydrological conditions (Fig. 12b).

(2) Cold Spots (Gi* < 0).

Conversely, cold spots indicate concentrated water area losses. Notable patterns include:

2014–2017 cold spots in central urban core suggest shrinkage of artificial water bodies amid urban densification (Fig. 10c). Persistent cold spots along the northeastern edge may reflect seasonal wetland drying or land reclamation (Fig. 12b).

To capture intra-annual variations in spatial clustering of water body changes, hot spot analysis was performed for each season during three time intervals: 2014–2017, 2017–2020, and 2020–2023.

In spring (Q1), significant hotspots were observed along the northern periphery of Zhengzhou, suggesting early-stage surface water restoration following winter contraction.

Summer (Q2) exhibited the most extensive clusters of water expansion, particularly in the Zhengdong New District and Huiji District, consistent with peak rainfall and wetland recharge.

Conversely, autumn (Q3) and winter (Q4) showed emergent cold spots in the urban core and northern periphery, indicative of seasonal drying and anthropogenic water withdrawal.

These temporal-spatial clusters provide insights into both hydrological and socio-environmental factors driving seasonal water dynamics.

#### Urban infrastructure correlation with hotspot–coldspot patterns

The spatial distribution of water body hotspots and coldspots identified through the Getis-Ord Gi* analysis shows a clear correlation with urban infrastructure transformation. The emergence of significant hotspots in the eastern and northeastern zones-notably in the Zhengdong New District-is aligned with the implementation of large-scale artificial lake construction, wetland ecological corridors, and river restoration projects initiated after 2017. Conversely, persistent coldspots in the central urban core overlap with areas of intense urban densification, where land reclamation, channel modification, and the filling of small ponds or ditches have contributed to continuous water body shrinkage. Additionally, southwestern clusters showing transition from coldspot to neutral or hotspot status may be associated with recent low-impact development initiatives. These patterns suggest that urban infrastructure planning plays a pivotal role in shaping the spatial dynamics of water body changes, either by promoting ecological recovery or by accelerating hydrological fragmentation in built-up zones.

#### Urban planning implications of hotspot–coldspot analysis

The results of the hotspot and coldspot analysis provide actionable spatial intelligence for future urban planning. Persistent hotspots-such as those in the Zhengdong New District and Yellow River floodplain-highlight regions where ecological interventions, such as wetland restoration and artificial lake development, have significantly enhanced water retention capacity. These areas can serve as model zones for replicable “blue-green” infrastructure strategies in other parts of the city. In contrast, coldspots concentrated in densely built-up districts reflect water body loss due to land reclamation and infrastructure expansion. These regions mays warrant policy interventions such as zoning adjustments, urban water-sensitive design, or retrofitting of small-scale ecological water features. By identifying where water bodies are thriving or declining, the spatial clustering results offer evidence-based guidance for balancing urban development with aquatic ecosystem preservation, which is critical for climate resilience, flood control, and sustainable urban growth in Zhengzhou.

### Discussion

The spatial evolution of water bodies in Zhengzhou from 2014 to 2023 reflects a coupled influence of climatic variability and intensive anthropogenic activities.

#### Impact of natural factors

Climatic variability plays a fundamental role in shaping the spatial and temporal patterns of surface water. During the study period, annual precipitation in Zhengzhou showed a generally increasing trend, with rainfall between 2017 and 2020 rising approximately 12% above the decadal average. This coincided with a 15% increase in total surface water area, indicating a strong correspondence between rainfall anomalies and water body expansion. Meanwhile, temperature trends suggest a gradual rise, which likely enhanced summer evapotranspiration and contributed to temporary water surface contraction.

Topographically, Zhengzhou’s terrain slopes from northwest to southeast. Water body changes were most pronounced in alluvial plains and low-lying zones prone to seasonal inundation. Spatial centroid analysis further revealed that newly added water surfaces exhibited an eastward shift of over 16 km during 2014–2023, closely following the regional slope and runoff direction.

#### Impact of human activities

Urban expansion has substantially altered land cover in Zhengzhou. From 2014 to 2023, built-up land increased by approximately 35%, replacing many small-scale water bodies such as irrigation ditches and fish ponds in peri-urban areas. Water loss hotspots were concentrated in zones between the second and third ring roads, where land conversion was most intense.

In contrast, policy-driven ecological initiatives promoted significant water restoration. Since 2020, the “blue–green infrastructure” campaign has led to wetland rehabilitation and artificial lake construction, especially in northeastern districts. Projects such as the Jialu River restoration and Longhu Eco-zone contributed to an estimated 8–10% increase in total water surface area in this sector alone, illustrating the impact of planned intervention on urban hydrology.

Together, these results demonstrate a coupled dynamic: precipitation surpluses amplify restoration effects, while urban densification exacerbates water loss in core areas.

#### Integrated analysis: drivers, spatial patterns, and future perspectives

Zhengzhou’s water body dynamics are shaped by short-term meteorological events and long-term land-use changes. For example, the sharp water surface increase in summer 2020-rising to 154.1 km²-was driven by an extreme rainfall event (260–350 mm in August), causing widespread flooding and rapid recharge of reservoirs and wetlands. Conversely, autumn 2023 saw notable contraction (to 169.3 km²) due to below-average precipitation and rising water demand. These event-driven shifts highlight the city’s hydrological sensitivity and underline the necessity of seasonally adaptive water management.

Spatially, hotspot and coldspot analysis reveals significant geographic patterns. Expansion hotspots align with newly developed districts like Zhengdong New District, where ecological engineering has improved water retention. Coldspots, in contrast, are concentrated in older central areas where small water bodies have been fragmented or lost. These trends are visual evidence of both successful planning and zones requiring urgent conservation.

Despite these findings, this study has limitations. The 30-meter resolution of Landsat 8 restricts detection of narrow or fragmented water features, especially in dense urban zones. Furthermore, with only four seasonal images per decade, rapid hydrological fluctuations may be underrepresented. Future work should incorporate higher-resolution optical (e.g., Sentinel-2, GF-2) and radar imagery (e.g., Sentinel-1 SAR) to improve spatial and temporal fidelity. Integrating auxiliary datasets-land use, precipitation time series, socioeconomic metrics-will also enable more robust modeling of urban hydrological responses. These efforts will elevate both scientific accuracy and policy relevance for water resource planning in fast-urbanizing regions.

## Conclusions

This study provides a comprehensive analysis of the spatiotemporal dynamics of surface water bodies in Zhengzhou, China, over the past decade (2014–2023), based on multi-seasonal Landsat 8 imagery. By integrating spectral indices, GLCM-based texture features, and random forest classification, we accurately delineated seasonal water body distributions and quantified both intra- and inter-annual changes. The results reveal an overall increase in surface water area, with seasonal peaks typically occurring between July and September. Spatial expansion was most pronounced in the northeastern and eastern districts, particularly in Zhengdong New District, while water body shrinkage was more evident in central urban areas. These trends were driven by a combination of climatic variability-especially precipitation extremes-and intensive human activities such as urban development and ecological restoration.

The findings contribute meaningful insights for urban water resource management and spatial planning. Hotspot and coldspot analyses identified key zones of water surface gain and loss, providing guidance for ecological conservation and targeted interventions. Patterns of centroid migration and seasonal fluctuation offer evidence to inform adaptive water allocation, flood control strategies, and the design of blue–green infrastructure in monsoon-affected urban environments.

To further improve the spatial precision and thematic reliability of urban water monitoring, future research should incorporate higher-resolution and multi-source datasets. Optical sensors such as Sentinel-2 (10 m) and GF-2 (< 1 m) can enhance detection of narrow or fragmented water bodies, while SAR imagery (e.g., Sentinel-1) supports monitoring under cloudy conditions or during flood events^[39]^. Integrating meteorological variables, land use data, digital elevation models, and socio-economic indicators will facilitate a more comprehensive attribution analysis of spatial drivers. This multi-source approach will strengthen the analytical robustness and policy relevance of water body assessments in rapidly urbanizing inland cities.

## Data Availability

The remote sensing imagery used in this study is publicly available from the United States Geological Survey (USGS) EarthExplorer platform (http://earthexplorer.usgs.gov/).The vector boundary data for Zhengzhou City were obtained from the Geospatial Data Cloud (http://www.gscloud.cn/).Daily and monthly precipitation data from 2014 to 2023 were acquired from the China Meteorological Data Service Center (http://data.cma.cn).All processed datasets and analysis results generated during this study are available from the corresponding author upon reasonable request.
